# Nutrient-limited subarctic caves harbour more diverse and complex bacterial communities than their surface soil

**DOI:** 10.1186/s40793-022-00435-z

**Published:** 2022-08-08

**Authors:** Ana Sofia Reboleira, Kasun H. Bodawatta, Nynne M. R. Ravn, Stein-Erik Lauritzen, Rannveig Øvrevik Skoglund, Michael Poulsen, Anders Michelsen, Knud Andreas Jønsson

**Affiliations:** 1 Departamento de Biologia Animal, and Centre for Ecology, Evolution and Environmental Changes (cE3c) & CHANGE - Global Change and Sustainability Institute, Faculdade de Ciências, Universidade de Lisboa, 1749–016 Lisbon, Portugal; 2 Natural History Museum of Denmark, University of Copenhagen, Universitetsparken 15, 2100 Copenhagen East, Denmark; 3 Department of Earth Science, University of Bergen, Allegt. 41, 5007 Bergen, Norway; 4 Department of Biosciences, Centre for Ecological and Evolutionary Synthesis (CEES), University of Oslo, 0316 Oslo, Norway; 5 Department of Geography, University of Bergen, Fosswinckels gt. 6, 5007 Bergen, Norway; 6 Section for Ecology and Evolution, Department of Biology, University of Copenhagen, Universitetsparken 15, 2100 Copenhagen East, Denmark; 7 Section for Terrestrial Ecology, Department of Biology, University of Copenhagen, Universitetsparken 15, 2100 Copenhagen East, Denmark

**Keywords:** Subterranean ecosystems, Subsurface, Subarctic ecosystems, Cave microbiomes, Microbial co-occurrence networks

## Abstract

**Background:**

Subarctic regions are particularly vulnerable to climate change, yet little is known about nutrient availability and biodiversity of their cave ecosystems. Such knowledge is crucial for predicting the vulnerability of these ecosystems to consequences of climate change. Thus, to improve our understanding of life in these habitats, we characterized environmental variables, as well as bacterial and invertebrate communities of six subarctic caves in Northern Norway.

**Results:**

Only a minuscule diversity of surface-adapted invertebrates were found in these caves. However, the bacterial communities in caves were compositionally different, more diverse and more complex than the nutrient-richer surface soil. Cave soil microbiomes were less variable between caves than between surface communities in the same area, suggesting that the stable cave environments with tougher conditions drive the uniform microbial communities. We also observed only a small proportion of cave bacterial genera originating from the surface, indicating unique cave-adapted microbial communities. Increased diversity within caves may stem from higher niche specialization and levels of interdependencies for nutrient cycling among bacterial taxa in these oligotrophic environments.

**Conclusions:**

Taken together this suggest that environmental changes, e.g., faster melting of snow as a result of global warming that could alter nutrient influx, can have a detrimental impact on interactions and dependencies of these complex communities. This comparative exploration of cave and surface microbiomes also lays the foundation to further investigate the long-term environmental variables that shape the biodiversity of these vulnerable ecosystems.

**Supplementary Information:**

The online version contains supplementary material available at 10.1186/s40793-022-00435-z.

## Background

Global warming threatens ecosystems worldwide through the disruption of natural weather cycles and ecosystem dynamics [1]. These effects are exacerbated at high latitudes where ecosystem dynamics are tightly linked to natural freezing and thawing cycles [2]. Our knowledge of subarctic ecosystems and their responses to disrupted annual climatic cycles is growing [3], but the knowledge of underground ecosystem dynamics, such as in caves, has remained largely neglected. Cave ecosystems differ substantially from surface ecosystems due to the lower diurnal and seasonal variation in temperature, and their higher humidity [4]. Darkness prevents photosynthesis, and primary production is absent in caves and aquifers, except for some unusual cases of chemolithoautotrophy [5]. Consequently, subterranean ecosystems presumably depend on organic matter transported from the surface to maintain heterotrophic productivity [6, 7]. Dissolved or particular organic matter comes from allochthonous sources, e.g., through water percolation, animal and wind transportation or through root penetration in superficial caves [6, 7]. The volume of organic matter and the form in which it enters will thus depend on cave connections and proximity to the surface; however, the influx is generally sporadic with high temporal variation [6], leading most caves to be relatively nutrientpoor (oligotrophic) [7].

Due to the unique abiotic characteristics and dependencies on organic matter input from the surface, life in subterranean environments is often accompanied by dramatic morphological, anatomical and ecological adaptations, and worldwide many species have evolved to be endemic cave specialists [8]. Furthermore, trophic chains in caves are generally regarded as simpler and with communities being characterised by (*i*) lacking photosynthetic primary producers, (*ii*) invertebrates that are typically adapted and confined to all aspects of their life cycle underground, and (*iii*) metabolically active microbes with important biogeochemical activities [9]. While the species diversity of cave macro-organisms is low [10], the diversity of cave micro-organisms can be as high as in surface communities [11–19].

Subarctic caves are characterized by a lack of cave-adapted invertebrates, likely as a result of recent glacial conditions [10]. Studies remain scarce but a few have demonstrated that such caves are inhabited by a low number of insect species that mainly belong to surface ecosystems (i.e., trogloxene insects [20, 21]), while studies of the microbial diversity of subarctic caves are limited to particular bacterial taxa carried out in the pre-omic era [22]. Surface bacterial communities in subarctic regions play a key role for soil structure and composition, providing a powerful tool to predict ecosystem responses to climate change [23]. Although some microbiomes have been characterized in cave habitats across the globe [11, 24–26], the lack of studies on bacterial communities in subarctic caves prevents us from knowing how these microbial communities function or whether and how they will be affected by global warming. The stability within caves can also be disrupted significantly by minor changes in temperature rises or increased influx of nutrients due to changes of the ecosystems above the ground [4]. Thus, it is important and timely to improve our knowledge of the biodiversity and nutrient cycling of subarctic caves to better understand the ecosystem dynamics in these fragile environments.

**Fig. 1:**
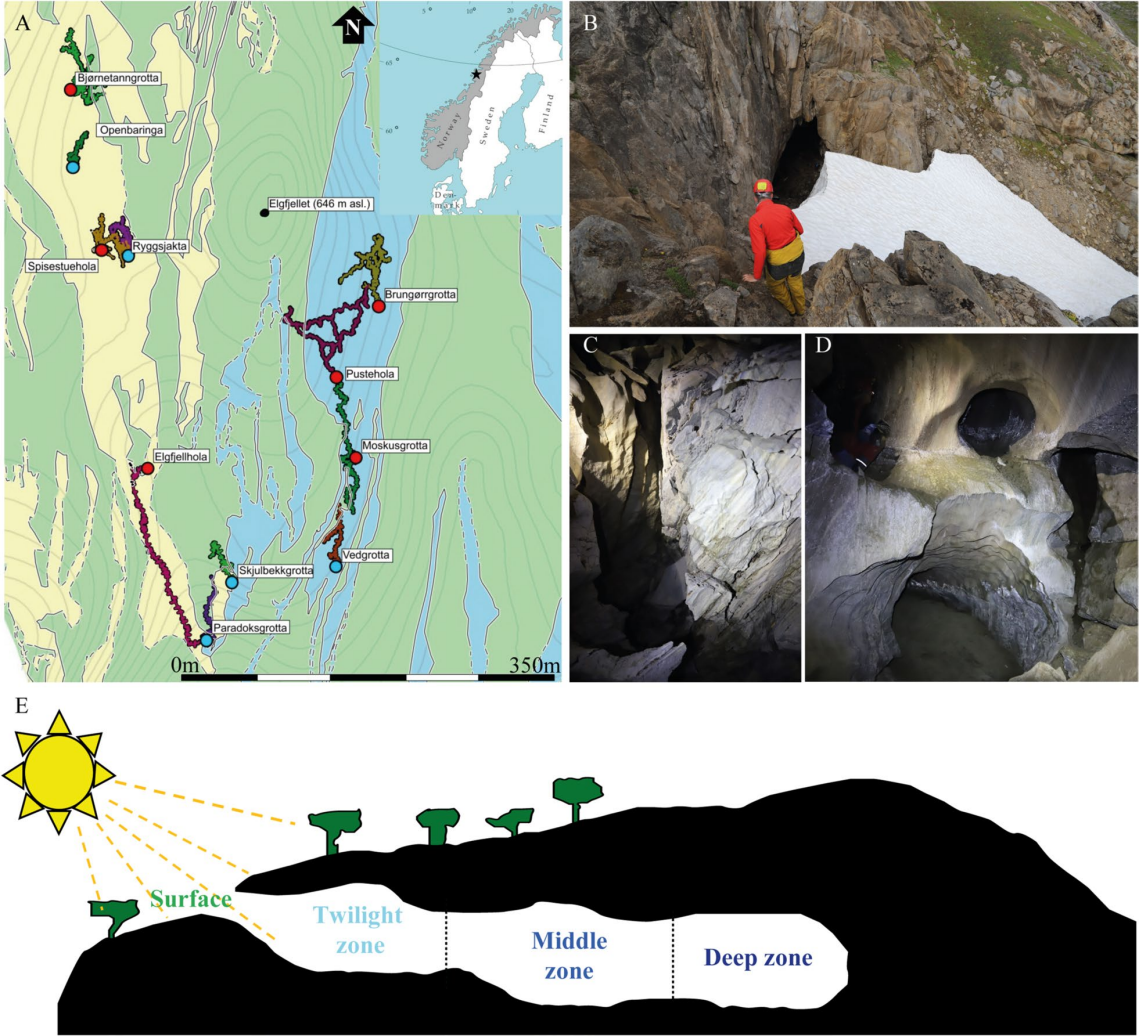
Exploration of invertebrate and bacterial diversity in six subarctic caves in Elgfjellet, Northern Norway. **A** Geographical map indicates the location of study site and depicts all the caves found in the study area, sampled caves are indicated with red dots. The colours on the geological map indicate different types of rocks that form the area (green: mica schist, blue: pure calcite marble, yellow: Mg- and mica rich marble). Images show examples of cave entrance (surface) (**B**), twilight zone (**C**), and inside the cave (**D**). **E** Schematic illustration of cave sampling zones investigated in the study

As a step towards doing so, we characterized biotic and abiotic parameters in six subarctic caves in Northern Norway (Fig. 1). We identified cave invertebrates and characterised bacterial community compositions and abiotic soil parameters for three zones within each cave: (i) the twilight zone, (ii) the middle zone and (iii) the deep zone. We compared these to the surface zone immediately outside caves (Fig. 1). We hypothesised that soil nutrient content would be highest at the surface and reduce as one moves through the twilight zone and the middle zone to the deepest zone and expect that microbial diversity is positively correlated with soil nutrient levels. Further, we hypothesised that bacterial communities would be more similar and stable between caves than surface sites of the same geographical area, with a major proportion of cave bacteria being unique to the cave environments.

## Methods

### *Localities and sampling*

We conducted fieldwork in six subarctic caves (Fig. 1; Additional file 1: Table S1) from August 4th–10th, 2019. The caves are located in Elgfjellet (“Moose Mountain”) at an elevation of 600–650 m a.s.l. in Nordland County in Norway just South of the Arctic Circle (Fig. 1 and Additional file 1: Table S1). The caves are mostly shallow, with the deepest penetration about 39 m below the surface (*Ryggsjaktene-Spisestuehullet*). Cave fills are glaciogenic silts and sands, moraine, with organic soil penetrating through grikes into passages at shallow depths. The karst rocks consist of narrow, N-S trending bands of Caledonian marbles, making the karst of the “stripe” type [27]. Elgfjellet displays a high density of caves [28], now incorporated into the Lomsdal-Visten National Park. Preliminary dating (M. Torstad, unpublished) suggests that these caves are older than the last interglacial, MIS-5, at 120 ka, in accordance with the general trend for caves within the Norwegian stripe karst [27, 29], where speleothems date back beyond 750 ka. It is therefore probable, that karst voids and caves have existed in the area for more than a million years.

The area is within the alpine zone, with bare, glaciated rock surfaces, sporadic moss and lichen cover and shrubs growing in depressions (e.g., *Betula nana*, *Betula pubescens* var. *pumila*, mountainous *Salix*, etc.). The tree-line appears at about 450–500 m a.s.l. During the Holocene climatic optimum (10–8 ka), as extrapolated from nearby areas [30], summer temperatures were about 2 ºC higher, and the tree-line probably 300–400 m higher than today. At that time, the area would have been covered with forest. Extrapolated from a nearby meteorological station (1967–1997 normal period, Majavatn, UTM N-722782 E-423500, 339 m a.s.l.), with an adiabatic lapse rate of 0.6 ºC/ 100 m, and a snow depth increase of 5 cm/100 m, the mean annual temperature is close to 0 ºC (max 14 ºC (July), min − 15 ºC (January), with a mean annual snow depth of 2.6–2.7 cm, and mean annual precipitation of 1200 mm.

We collected sediments aseptically in 0–3 cm depth for each cave at four sites: (i) at the surface immediately outside the cave, (ii) in the twilight zone, (iii) in the middle zone, and (iv) in the deepest zone (Fig. 1) and samples were kept cold until reaching the lab for processing. We characterized invertebrate fauna using baited pitfall traps and by actively searching in the deepest parts of the caves for five days. The pitfall traps were baited with Norwegian caviar and 1,2-propanodiol was used for preservation. All specimens have been deposited in the Natural History Museum of Denmark.

### *Environmental variables*

We measured the temperature inside the caves with dataloggers TidbiT v2 Temp UTBI-001 that were placed in the deepest part of each cave, recording temperatures every 2 h. Temperature data were downloaded through an Optic USB Base Station (BASE-U-4) using the HOBOware Software. To remove large roots from the soil samples, we sieved (2 mm) each sample from the four zones of the six caves individually and mixed carefully. Each fresh soil sample was divided into five subsamples (four for nutrient analyses and one for microbiome analysis). Using these soil samples, we characterized soil pH, water content, soil organic matter (SOM), carbon and nitrogen proportions, dissolved organic carbon (DOC) and nitrogen (DON), inorganic nitrogen content, phosphate (${\text{PO}}_{4}^{ - 3}$), nitrate (${\text{NO}}_{3}^{ - }$), and ammonium (${\text{NH}}_{4}^{ + }$) ion contents, soil conductivity, and microbial bound carbon (MicC), nitrogen (MicN) and phosphorous (MicP) contents.

We measured the pH (pHM240 MeterLab) and conductivity (SevenCompact Conductivity) of soil samples through suspending one subsample (10 g) of fresh soil in demineralized water (ratio 1:5). We used another subsample to analyze microbial C (MicC) and microbial N (MicN) using the chloroform fumigation method [31, 31]. To do this, we first suspended 20 g of soil for one hour in demineralized water (ratio 1 g soil:5 ml H_2_O) and filtered using Whatman GF/D. Another 20 g subsample was incubated for 24 h in a vacuum desiccator with chloroform before extraction and filtration. All filtrated extractions were kept frozen until analysis.

Samples were thawed and centrifuged for 10 min at 3,161 rcf (4,200 rounds per minute—rpm). Hundred mL of 2 M HCL was added to an extraction of non-fumigated soil and 50 μL of 2 M HCl was added to an extraction of fumigated soil before freeze-drying. All material from each freeze-dried sample was packed individually in tin capsules and analyzed on an isotope ratio mass spectrometer (IRMS; Isoprime) connected to an Eurovector CN elemental analyzer to determine the total dissolved C (TDC) and the total dissolved N (TDN). Estimation of MicC and MicN was based on the difference between fumigated and non-fumigated samples using an extractability factor of 0.45 for C and 0.40 for N [33–35]. Isotope ratios of microbes are based on values from fumigated soil. Furthermore, the extracts of non-fumigated soil were analyzed for ${\text{NO}}_{3}^{ - }$ and ${\text{NH}}_{4}^{ + }$ content using flow injection analysis (FIAstar 5000 Analyzer).

To determine soil water content, the remaining soil from each sample was dried at 60 °C for three days. Subsequently, the dry soil was ground in a ball mixer and approximately 10 mg of soil was packed in tin capsules and analyzed on IRMS to determine the concentrations of C and N.

### *Bacterial community characterisation*

We extracted DNA for microbiome analyses from 0.25 g of soil in triplicates from each soil sample, using the Qiagen DNeasy® PowerSoil® kit (Hilden, Germany) following the manufacture’s guidelines. Initial PCRs to identify samples with bacterial DNA were conducted using  two bacteria-specific primers targeting the V4 region of the 16S rRNA (SB711 and SA504) and following a well-establish protocol for the primers [36]. All samples amplified and were sent to the Microbiome Core at the University of Michigan for MiSeq amplicon sequencing on an Illumina platform.

We analysed bacterial sequences in Qiime2 [37] using the DADA2 pipeline [38]. Amplicon sequence variants (ASVs) at 100% similarity were taxonomically assigned using the SILVA 132 bacterial reference library [39]. Subsequently, archaeal, mitochondrial and chloroplast sequences were removed. We acquired the bacterial phylogenetic tree using the ‘align-to-tree-mafft-fasttree' function in QIIME2 [37]. Overall, the microbial communities of triplicates were similar within sampling localities based on visual inspection of ordination plots generated using Bray–Curtis distances (Additional file 1: Fig. S1, Additional file 2: Table S2). Thus, for further analyses, we averaged the data from triplicates and removed any ASVs containing less than 20 total sequences (Additional file 3: Table S3). Due to differences in sequencing depth across samples (average ± SD: 18,049 ± 3298 SD), we rarefied the original dataset using the sample with the smallest number of sequences (10,767) using the rarefy-even-depth function in phyloseq package [40]. Subsequent analyses were conducted on the rarefied dataset, unless specified.

### *Statistical analysis*

All statistical analyses were performed in R 4.0 and RStudio [41]. For soil properties, we first tested the variance of homogeneity using Levene’s test and data were log or square root transformed prior to further analyses in cases of heterogeneous variance . Differences in environmental variables between sample zones (surface, twilight, middle and deep) were tested using one-way analysis of variance (ANOVA) and Tukey’s test. If variance of heterogeneity persisted, nonparametric tests (Kruskal Wallis—KW) and Dunn’s post-hoc tests were used with the FSA [42] and the dplyr [43] packages. We considered *p* values < 0.05 significant, but we also report tendencies towards significance (0.05 < *p* < 0.10). Correlations between soil environmental variables were performed using Pearson’s correlation.

Alpha diversities of bacterial communities (observed ASV richness, Chao 1 richness and Shannon’s diversity index) were estimated using the phyloseq package [40]. We also calculated core microbial dominance (the proportion of ASVs with > 2% relative abundance in > 50% of samples) using the microbiome package [44], to investigate whether microbial communities in different cave zones are dominated by few or more bacterial taxa. KW tests and Dunn’s post-hoc tests were utilized to investigate the statistical differences. Finally, we used linear regressions to statistically test associations between bacterial ASV richness and soil properties (e.g., pH and water content).

Bacterial community level differences were visualized using non-metric multidimensional scaling (NMDS) plots using Bray–Curtis distances. The environmental variables that were significantly associated with community level differences were identified using envfit function in vegan [45]. Differences in microbial communities (based on Bray–Curtis distances) in different sampling zones of the caves were examined with permutational multivariate analysis of variance (PERMANOVA with 10,000 permutations) using the adonis function in the vegan package [45] and pairwise differences were explored using the wrapper package pairwiseAdonis [46].

We identified significantly differentially abundant bacterial taxa between soil microbial communities in different cave zones using linear discriminant analysis effect size (LEfSe) [47]. KW tests along with linear discriminant analyses (LDA > 3.5) were used to determine differentially abundant features using the microeco package [48]. Furthermore, to assess associations between different ASVs in bacterial communities in different cave zones we generated microbial co-occurrence networks using the SpiecEasi [49] and the pulsar [50] packages. Here we used the original dataset as the SpiecEasi conduct internal normalisation of bacterial communities. The networks were only generated for taxa with > 100 sequences using the Meinshausen and the Bühlmann neighbourhood selection method with 600 repetitions (subsamples). Correct model sparseness was inferred using the Stability Approach to Regularization Selection (StARS) criterion [51] and lambda parameters were adjusted until the stability of the network was close to a 0.05 threshold [49]. The output network files were used to investigate network properties such as number of nodes (how many ASVs are interacting with one another), positive and negative interactions, number of interactions ASVs have with one another (degree), number of distinct communities within the network, the strength of community division (modularity), and network assortativity using igraph package [52]. The network assortivity provide insights into whether ASVs with similar degree (similar number of associations with other ASVs) interact with one another. This coefficient varies from −1 to + 1, where negative values represent a network with ASVs with large degree interact with ASVs with smaller degree and positive values indicate ASVs with similar degree interact with each other.

To investigate whether bacterial communities within cave zones originate from the surface, we conducted a source tracking analysis using the SourceTracker 1.0 [53]. We conducted these analyses at the bacterial genus level and labeled all surface samples as “source” and cave zones as “sinks”. We trained the dataset using these source-sink categories and predicted the proportion of bacterial genera within cave zones that might have originated from the surface. Additionally, to investigate the role of environmental filtering and environmental change on structuring microbial communities, we investigated the nearest taxon index (NTI) and the phylogenetic beta diversity by calculating the beta nearest taxon index (βNTI) in each zone [54]. These indices use the standardized effect size of the mean nearest taxon distance (MNTD) to calculate the mean phylogenetic distance in taxa within communities (NTI) and between different communities (βNTI). NTI values bigger than + 2 indicate communities with lower phylogenetic diversity than expected by chance while in βNTI, this indicates lower phylogenetic turnover among communities than expected by chance (coexistence of phylogenetically related taxa between communities). Values smaller than −2 indicate phylogenetically diverse microbial communities (for NTI) and strong phylogenetic turnover among communities (for βNTI). These indices can be used to evaluate the influence of environmental filtering and environmental change on structuring microbiomes [54]. Thus, if environmental filtering is a strong driver of communities within caves, we expected the coexisting taxa to be more closely related than null model predictions (2 < NTI). Second, if the environmental change between surface and caves drives bacterial community compositions, we would expect greater phylogenetic turnover (βNTI < −2) between surface localities compared to among cave zones. We calculated the NTI through negative conversion of the ses.mntd output from the picante package [55, 56]. We calculated the βNTI using the cal_ses_betamntd function in the microeco package [48]. For both NTI and βNTI we compared the observed values with 1,000 randomly generated communities.

## Results

### *Environmental variables differ between surface and cave zones*

**Fig. 2:**
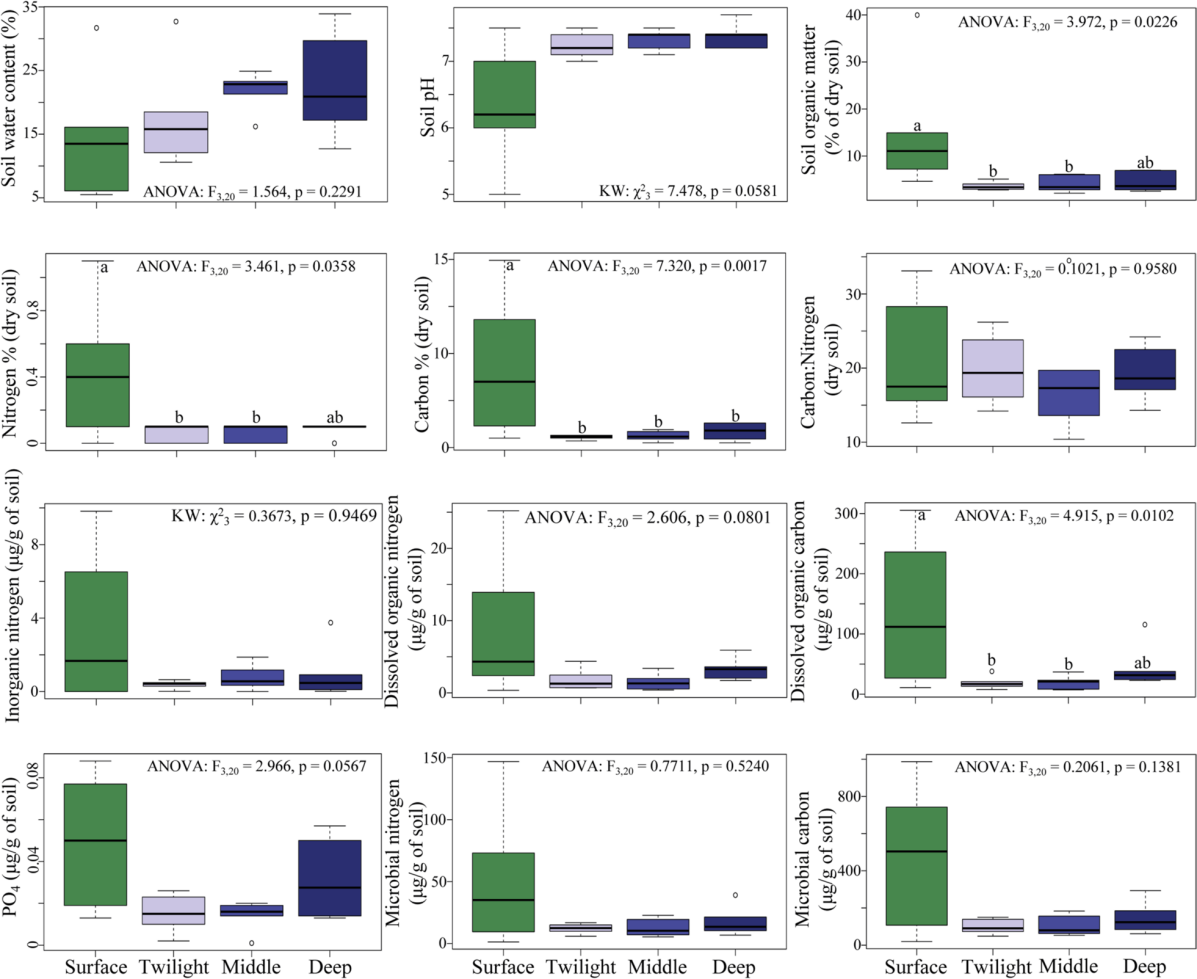
Abiotic factors differ between surface and cave environments but not across cave zones. Boxplots representing a subset of measured soil properties at the surface and in the three cave zones (twilight, middle and the deep zones) (Additional file 4: Table S4). Each individual boxplot of data represents analysis of six samples from different cave systems. Results of statistical tests are given within each graph and significant post-hoc results based on pair-wise comparisons between zones are indicated with different letters

Multiple soil properties differed between surface and cave zones, but interestingly these properties did not differ between zones within caves (Fig. 2, Additional file 4: Table S4 and Additional file 1: Table S5). Furthermore, we observed higher variation in properties in surface soils compared to soil zones in caves. We did not observe significant differences in water content between different soil zones, but on average there was more water in the middle and deep zones of caves, indicating potential higher levels of water retention or accumulation, probably due to higher atmospheric humidity. Soil pH was marginally significantly higher (more alkaline) in the cave zones compared to the surface (Fig. 2, Additional file 1: Table S5), a result of the high amount of carbonate minerals presents in cave rocks. In contrast, the content of SOM was higher in surface compared to cave zones (Fig. 2, Additional file 1: Table S5 and S6). This was expected as there is more assimilation of organic matter in surface habitats with higher plant, fungal and animal biomass. Aligning with this, we observed higher total carbon and nitrogen concentrations in the dry soils at the surface (Fig. 2, Additional file 1: Tables S5 and S6). However, the C:N ratio did not differ between zones (Fig. 2). Furthermore, DON tended to differ between zones (Fig. 2, Additional file 1: Table S5), while DOC levels differed significantly between zones (Fig. 2, Additional file 1: Table S5) with a higher concentration at the surface compared to middle and twilight zones (Additional file 1: Table S6).

We did not observe significant differences in conductivity (µS cm^−1^) of the soil sediment across sampling zones. Aligning with this, levels of different ions (e.g., ${\text{PO}}_{4}^{ - 3}$, ${\text{NO}}_{3}^{ - }$, ${\text{NH}}_{4}^{ + }$) did not differ between the zones (Fig. 2, Additional file 1: Table S5). However, the variation in conductivity among caves is high and is positively correlated with levels of ${\text{NH}}_{4}^{ + }$ (*p* = 0.0101, r = 0.5144), ${\text{NO}}_{3}^{ - }$ (*p* = 0.015, r = 0.4902), inorganic N (*p* = 0.0058, r = 0.5458) and tends to be correlated with ${\text{PO}}_{4}^{ - 3}$ (*p* = 0.0539, r = 0.3982).

The microbial biomass (bacteria, archaea, and fungi), MicC, MicN, and MicP, and microbial C:N ratios did not differ significantly between sampling zones (Additional file 1: Table S5). On average, there was a trend of higher levels of microbial bound elements in the surface soils, potentially reflecting a higher abundance of fungi at the surface—likely mycorrhizal fungi and fungal taxa associated with lichens (Fig. 2). However, these values showed a high variation in surface soils compared to the cave zones, indicating a high variation in microbes in surface soils of subarctic regions (Fig. 2).

### *Lack of cave-adapted invertebrate fauna*

Consistent with previous works [20, 21], the invertebrate community was composed of tardigrades and arthropods (arachnids and insects), all considered trogloxenes, i.e., surface-adapted animals (Additional file 1: Table S7). We only captured a total of 89 invertebrates (average ± SD: 15 ± 18) from the six caves (Additional file 1: Table S7). The cave with the highest abundance was the Bjørnetanngrotta cave with 48 individuals. The most abundant species was *Trichocera regelationis* (order Diptera) with 68 individuals, corresponding to 76.4% of all captured invertebrates, and they were found in five out of six caves (Additional file 1: Table S7).

### *Surface bacterial communities were less diverse than cave communities*

**Fig. 3:**
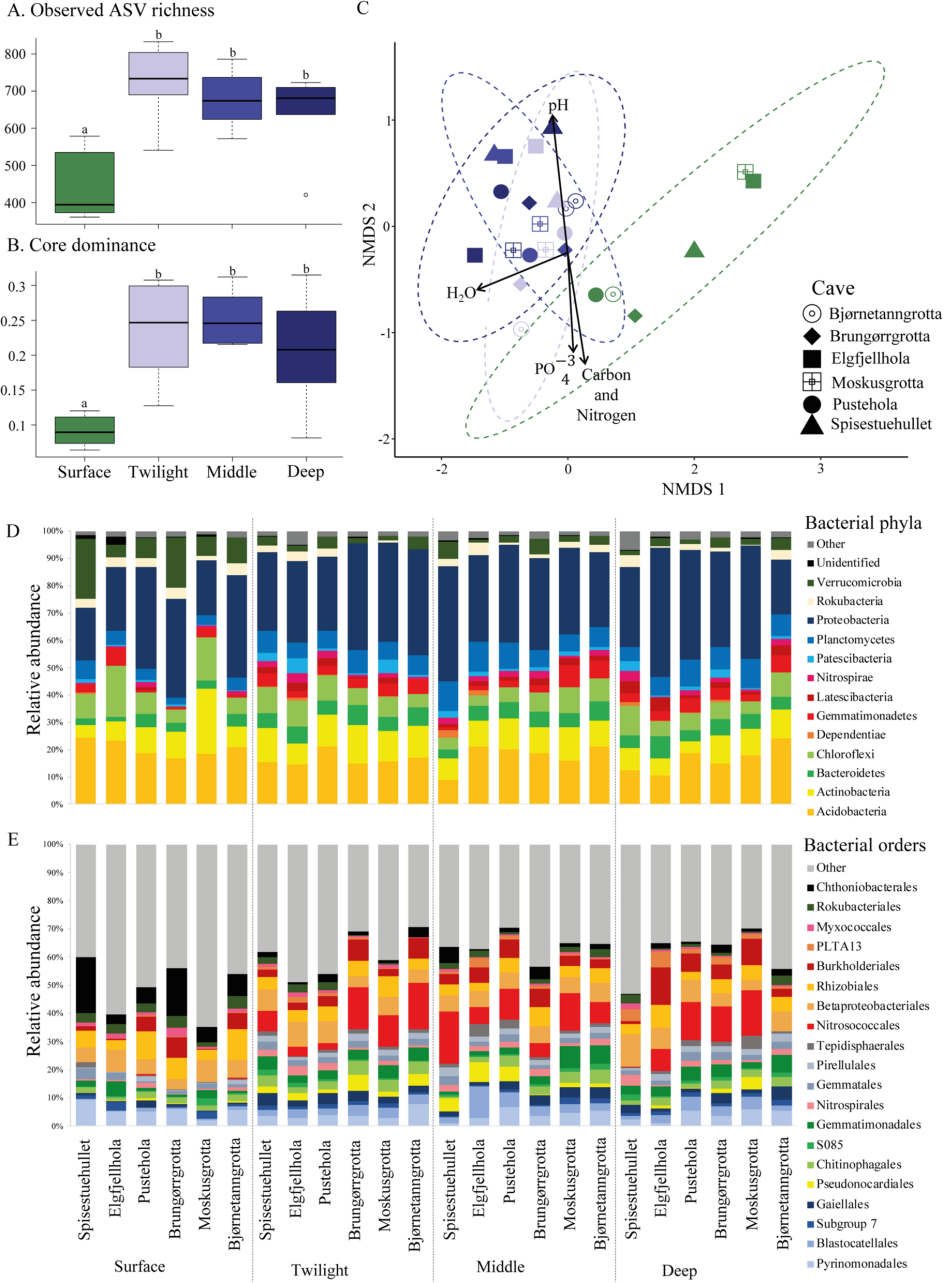
Cave microbial communities are richer, harbour more abundant bacterial taxa and are phylogenetically distinct from surface communities. ASV richness (**A**) and core dominance (proportion of ASVs with > 0.2% relative abundance across > 50% of the samples) (**B**) across cave zones. Post-hoc results based on pair-wise comparisons between soil zones are indicated with letters. **C** NMDS plot showing bacterial community similarities (Bray–Curtis distances) among sampling zones and caves (stress = 0.0701). Colours of sampling zones are the same as the colours in panels A and B. Arrows indicate the four environmental variables that were significantly associated with the observed differences in bacterial community compositions. Ellipses represent the 95% confidence intervals. **D** Relative abundance of major bacterial phyla and the 20 most abundant bacterial orders (**E**) across sampling zones

Prior to pooling triplicates of microbial communities from the same sampling site, we acquired 1,452,155 sequences (20,169 per sample ± 453 SE) (Additional file 2: Table S2). After removal of low abundant ASVs and averaging the triplicates, we acquired a total of 426,107 sequences (17,754 per zone in a cave ± 670.07 SE) assigned to 3,004 ASVs (Additional file 3: Table S3). Average observed ASV richness (KW χ^2^ = 12.31, df = 3, *p* = 0.0064) and Chao 1 richness estimates (KW χ^2^ = 12.83, df = 3, *p* = 0.0051) were significantly lower for surface soil communities than any of the cave zones (Fig. 3A, Additional file 1: Table S8). There was no significant difference in ASV richness between cave zones (Additional file 1: Table S8). Shannon’s diversity index did not differ among any of the soil communities (KW χ^2^ = 3.247, df = 3, *p* = 0.3551). The core dominance (the proportion of ASVs with > 0.2% relative abundance in > 50% samples) was significantly lower in the surface soil communities compared to the cave communities (KW χ^2^ = 12.35, df = 3, *p* = 0.0063, Fig. 3B and Additional file 1: Table S8), indicating that fewer bacterial ASVs dominate the surface soil communities compared to in the cave (higher number of abundant bacterial taxa within caves).

### *Bacterial richness was associated with some soil properties*

**Fig. 4:**
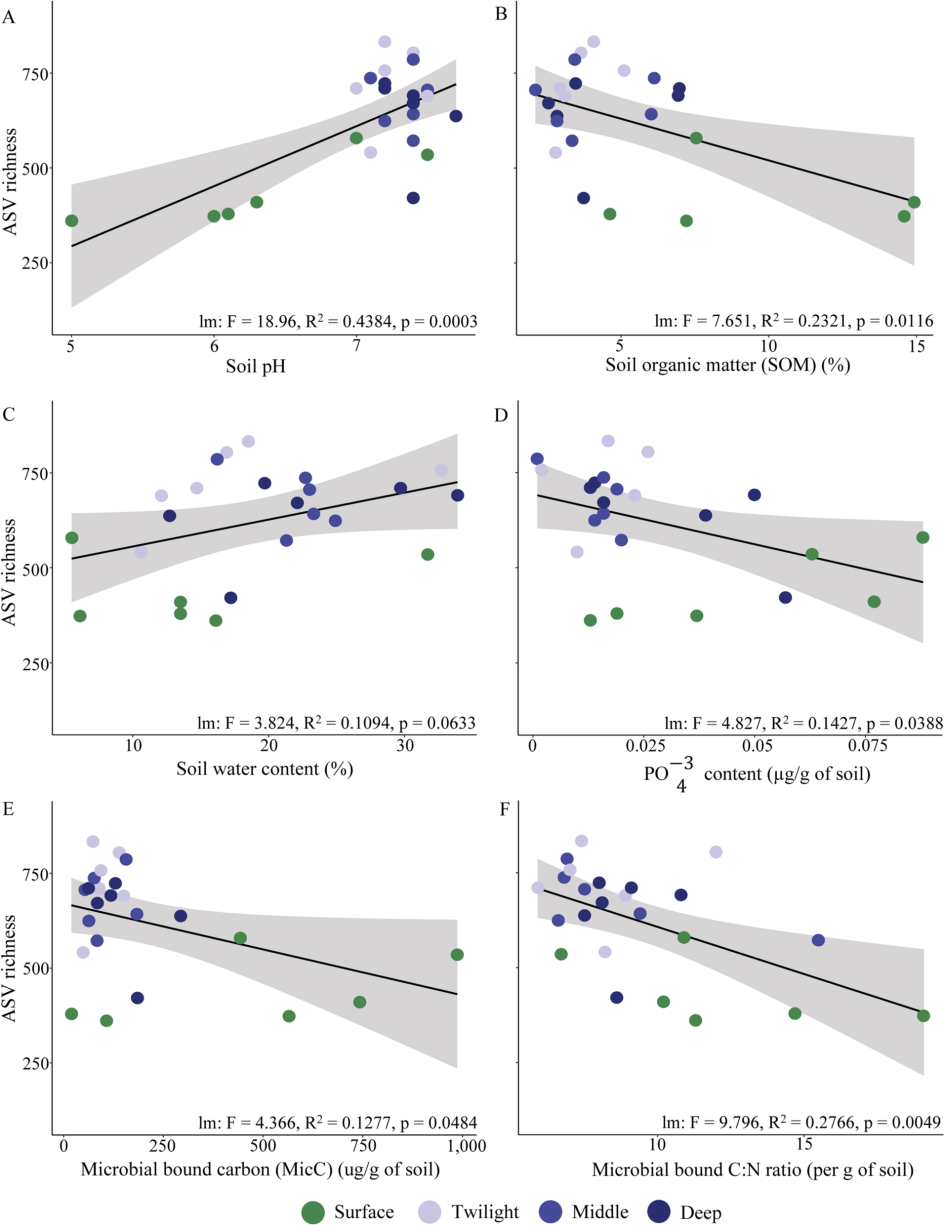
Soil bacterial richness was associated with multiple soil properties. Relationships between bacterial ASV richness and six selected soil properties (**A**. pH, **B**. SOM, **C**. water content, **D**. ${\text{PO}}_{4}^{ - 3}$ content, **E**. MicC and **F**. microbial C:N, full results can be found in Additional file 1: Table S9). Results of linear models are given for each regression analysis within each graph and standard errors are indicated as grey shaded areas around the trend lines

Multiple soil parameters tended to be associated with bacterial richness (Additional file 1: Table S9), of which we observed a few significant associations (Fig. 4). Most prominently, ASV richness was significantly positively associated with pH, and increased water content tended to lead to richer bacterial communities (Fig. 4 and Additional file 1: Table S9). Of the soil nutrient parameters, ASV richness was significantly negatively associated with ${\text{PO}}_{4}^{ - 3}$ concentration (Fig. 4) and close to significantly negatively associated with levels of SOM, nitrogen content, carbon, DON, DOC and ${\text{NH}}_{4}^{ + }$ (Additional file 1: Table S9). Overall, this suggest that cave soils with reduced nutrient levels (Fig. 2) tend to harbour more diverse bacterial communities. Despite the higher bacterial richness in cave environments, we found a significantly negative association between ASV richness and levels of MicC and microbial C:N ratio (Fig. 4). Measurements of microbial bound elements also capture other microbes such as archaea and fungi, thus this observed association might be influenced by the higher fungal biomasses found in surface communities, which also is consistent with the fact that high microbial C/N ratios generally reflect relatively higher fungal dominance [34].

### *Community composition of surface microbiomes differ from cave zones*

Bacterial communities differed significantly between the surface and all cave zones combined (PERMANOVA_10,000 permutations_: F_3,23_ = 2.472, R^2^ = 0.2635, *p* < 0.0001: Fig. 3C and Additional file 1: Table S10). However, the bacterial communities among the three cave zones did not differ significantly from each other. The observed differences between the surface communities and the three cave zones were strongly linked with increasing pH and water levels and reducing soil nutrients (Fig. 3C, Additional file 1: Table S11).

We identified 33 bacterial phyla, to which 99.6% of bacterial sequences were assigned, while 81.2% could be assigned to an order and 63.0% to a family. Overall, the relative abundance of Proteobacteria dominated soil communities (average ± SD: 32.9% ± 7.5%), followed by Acidobacteria (17.8% ± 4.0%), Actinobacteria (10.0% ± 3.8%), Chloroflexi (7.9% ± 3.5%) and Planctomycetes (7.0% ± 2.3%) (Fig. 3D and E, Additional file 1: Table S12). Both surface and cave zone bacterial communities were dominated by the same main bacterial phyla (e.g., Proteobacteria, Acidobacteria and Actinobacteria). However, the relative abundance of some bacterial phyla, such as Verrucomicrobia (surface: 11.5% ± 7.0% vs. cave: 3.1% ± 1.5%), Chloroflexi (surface: 10.3% ± 5.6% vs. cave: 7.1% ± 2.1%) and Rokubacteria (surface: 3.4% ± 1.0% vs. cave: 2.0% ± 1.4%) were higher at the surface compared to cave zones, while the relative abundance of bacterial phyla, such as Planctomycetes (surface: 4.4% ± 1.5% vs. cave: 7.9% ± 1.8%), Nitrospirae (surface: 0.8% ± 0.7% vs. cave: 1.9% ± 1.0%) and Patescibacteria (surface: 0.8% ± 0.4% vs. cave: 1.9% ± 1.5%) were higher in the caves compared to the surface (Figs. 3D and E, Additional file 1: Table S12).

### *Abundances of multiple bacterial orders differed significantly between surface and cave zones*

**Fig. 5:**
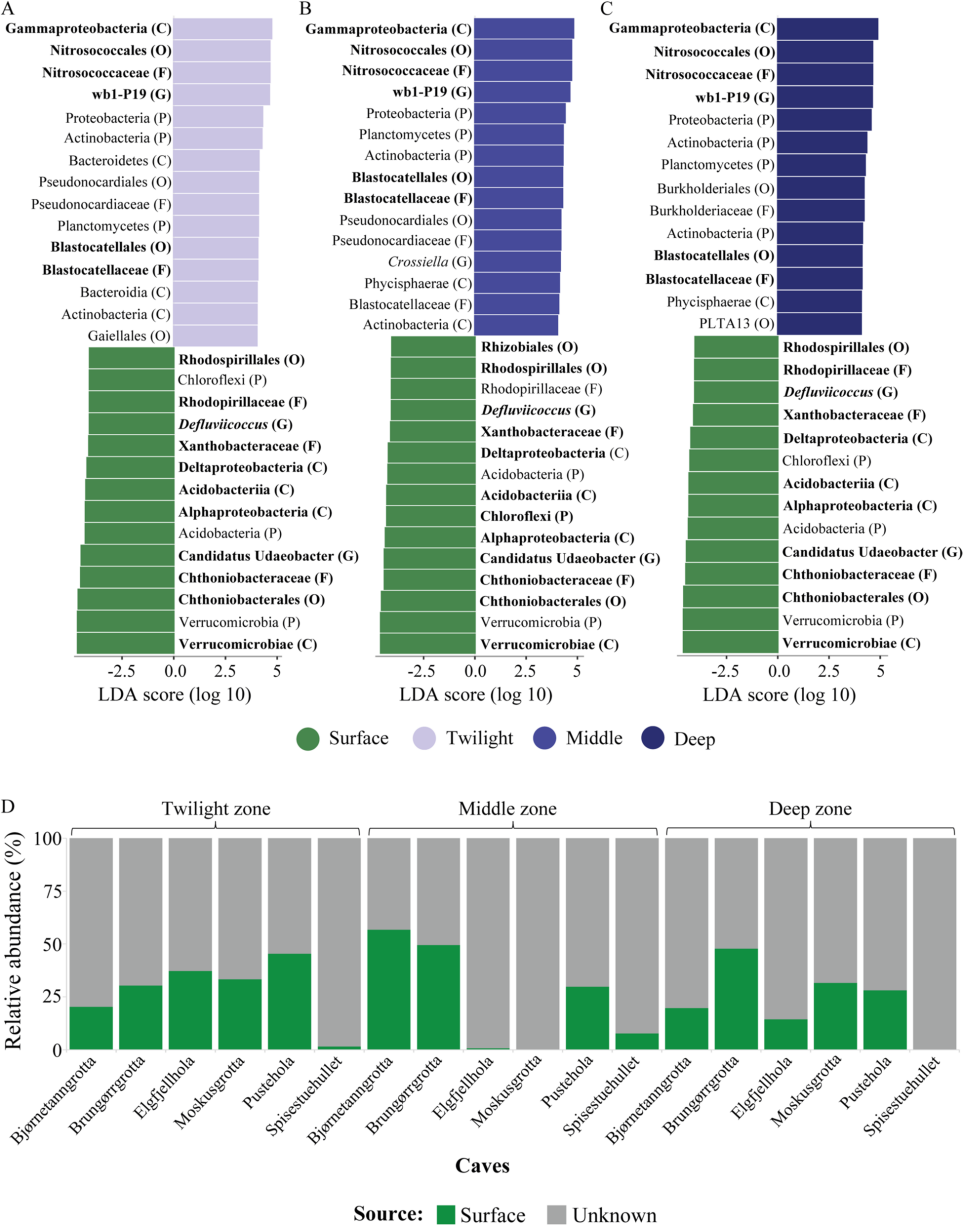
The same bacterial taxa contribute significantly to community level differences between surfaces and cave zones, and only a small proportion of surface bacterial genera are shared with cave zones. **A**-**C** Bacterial taxa that were significantly differentially abundant (LDA value > 3.5) between the surface communities and the three cave zones [twilight (**A**), middle (**B**), deep (**C**)]  based on LEfSe analyses. Taxa that were significantly (relatively) more abundant in the surface communities compared to communities in all three cave zones are highlighted in bold. The closest taxonomic-level identification of the taxa are indicated in parenthesis (P—Phylum, C—Class, O—Order, F—Family, G—Genus). **D**. The bar chart represents the proportion of bacterial genera in cave zones that were predicted to originate from surface communities based on the source tracking analysis

Our LEfSe analyses [47] identified which bacterial taxa significantly influenced the observed differences in bacterial communities between surface and cave soil zones. Since we only observed significantly different microbial communities between the surface soil and the three cave zones, and not between cave zones (Fig. 3C, Additional file 1: Table S10), we only conducted LEfSe analysis between surface vs. twilight zone, surface vs. middle zone, and surface vs. deep zone. Bacterial taxa that contributed to the differences between the surface communities and the three cave zones were often shared (Fig. 5A–C). The four most significantly differentially abundant taxa were identical across comparisons of surface soils to the three cave zones, and they belonged mainly to the phylum Verrucomicrobia (Fig. 5A–C). Unidentified taxa from the phylum Gemmatimonadetes and the order Nitrosococcales (phylum Proteobacteria) were significantly more abundant in the cave communities compared to in the surface communities (Fig. 5A–C).

### *Cave microbiomes have more complex associations between ASVs*

**Fig. 6:**
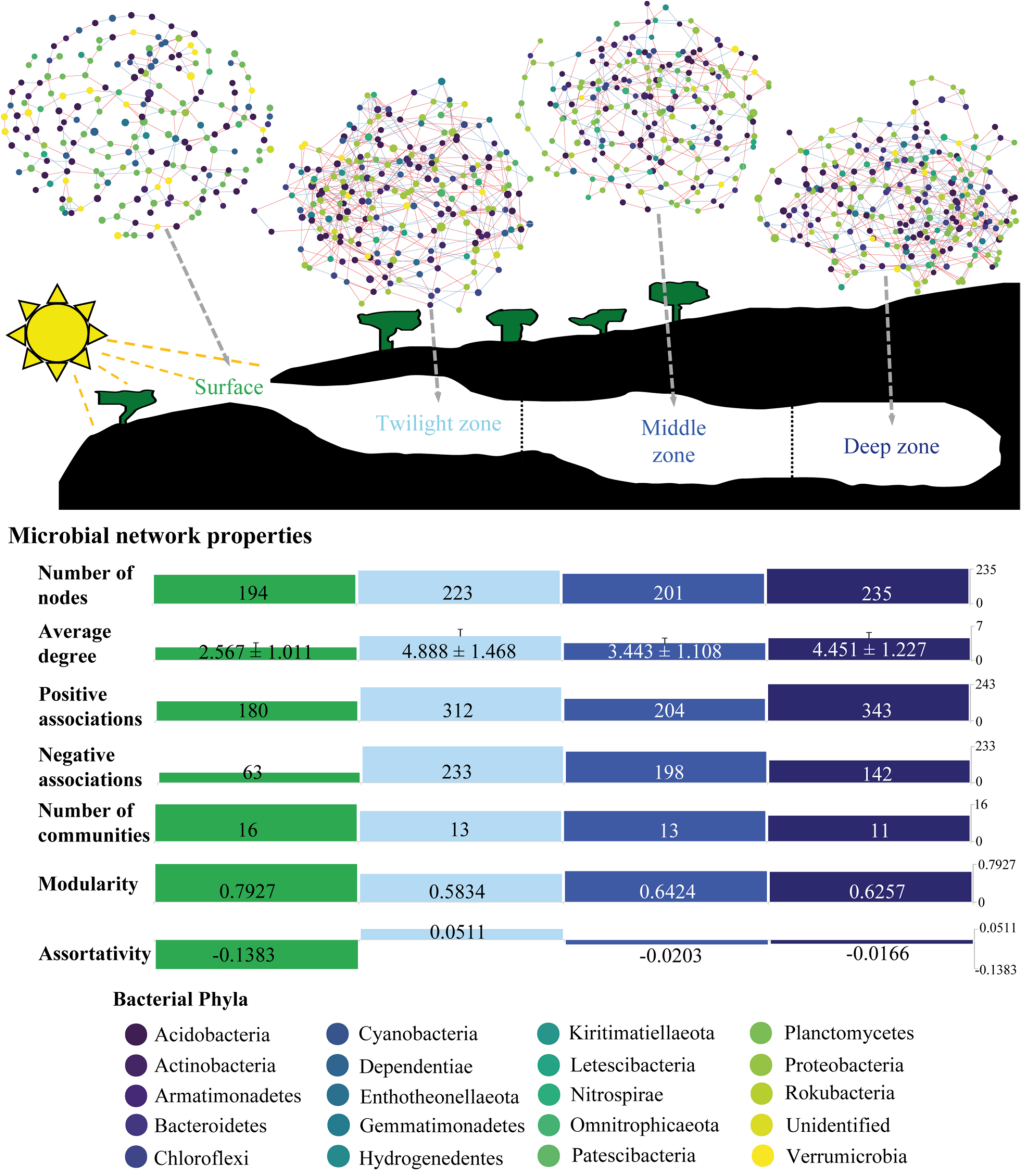
Microbial co-occurrence networks are modular with fewer associations between bacterial taxa in surfaces than in cave communities. Nodes represent ASVs and connections represent associations between different ASVs. The sizes of the nodes indicate the geometric mean of the relative abundance of each ASV and colours represent bacterial phyla of the ASVs. Edge colours show positive (red) and negative (blue) associations. The lower panels provide different network properties, including the number of nodes (the number of ASVs that have associations with other ASVs), average degree per node ± SD (average number of interactions one ASV has with others; the error bars indicate the standard deviations), number of positive and negative associations between ASVs, number of communities (optimal division of network into sub-communities), modularity (strength of community division), and assortativity (testing whether similar nodes are interacting with each other)

Bacterial co-occurrence networks harboured comparable number of nodes (ASVs) between different soil communities (Fig. 6). However, the networks within cave zones harboured more interactions between ASVs (high degree) and more positive and negative associations compared to surface communities (Fig. 6, Additional file 1: Fig S2), indicating more complex and interdependent microbial associations within caves compared to in surface soil. The proportion of positive associations were higher than negative associations in all zones, indicating that the abundance of many bacterial taxa in these subarctic soil communities depend on other bacterial taxa. Furthermore, surface bacterial network harboured more distinct ASV clusters within the network; hence being more modular than cave networks (Fig. 6). This suggests that bacterial communities are more compartmentalized in surface soils and potentially with a higher level of shared ecological functions than in cave communities [57]. Finally, we found more negative assortativity in the surface, suggesting that ASVs with a higher degree (more interactions with other ASVs) associate with ASVs with a low degree, compared to cave zones (Fig. 6).

### *Surface communities are not sources of cave bacterial communities and limited evidence for environmental filtering in cave microbiomes*

The source tracking analysis revealed that only a small proportion of bacterial genera in cave zones (average ± SD: Twilight zone: 28.0% ± 15%, Middle zone: 24.1% ± 25.0%, 23.6% ± 16.3%) can be predicted to have originated from the surface (Fig. 5D). This indicates that  cave-adapted bacterial taxa are dominating these cave microbiomes. However, the proportions of predicted surface-originated bacterial genera vary between caves, indicating different influx levels of surface bacteria into cave habitats. On average, we observed more positive NTI in cave zone communities compared to the surface, indicating that cave microbial communities are less phylogenetically diverse (KW χ^2^ = 1.981, df = 3, *p* = 0.1733: Additional file 1: Fig. S3A). However, they did not differ among cave zones (KW) nor had NTIs > 2, indicating these values do not deviate from random expectations, suggesting that environmental filtering might not have a strong influence on structuring these bacterial communities [54]. The phylogenetic community turnover (βNTI) was highly variable among surface communities compared to cave zones and was lower in cave zones compared to the surface, indicating reduced phylogenetic turnover among cave communities compared to across surface communities (KW χ^2^ = 14.61, df = 3, *p* = 0.0022: Additional file 1: Fig. S3B). However, the pair-wise comparisons revealed that only surface βNTI differed significantly from the deep zone. Similar to NTI, we did not observe βNTI values > +2 or < −2, indicating that phylogenetic community turnovers do not deviate from null expectations.

## Discussion

The transition from surface to subterranean ecosystems involves marked environmental changes, resulting from a combination of lack of light, low nutrient content and more stable conditions [9, 58]. Here, we investigated the invertebrate and bacterial communities, along with soil properties of understudied caves in subarctic Northern Norway to understand biotic interactions with nutrient availability in these fragile ecosystems. Invertebrate diversity was low, dominated by surface-adapted species (= trogloxenes), consistent with previous studies in the area [20, 21]. These invertebrates are unlikely to play significant roles for nutrient cycling in these ecosystems [59, 60]. In contrast, we observed diverse and complex bacterial communities, aligning with bacterial diversities observed in other cave systems [11, 24, 25]. As predicted, soil nutrient levels were higher at the surface than in caves, but we did not find a gradient in nutrient reduction when moving from the twilight zone to the deeper zones; thus, we only found partial support for our hypothesis that nutrient flow is highly limited in all cave zone. Also contradicting our hypothesis, we found that bacterial diversity was negatively associated with several soil variables and higher in nutrient-poor caves than in nutrient-rich surface soils. We did, however, confirm our prediction that cave communities were consistent across cave zones and different from surface soil, with limited influx of bacterial taxa from surfaces to caves.

### *Distinct abiotic conditions and soil characteristics within caves shape bacterial communities*

The positive association of increased pH in caves with both alpha and beta bacterial diversity aligns with previous studies [23, 61], including in other cave systems [26]. Finding richer bacterial communities with decreasing soil nutrient content was, however, unexpected, as a study on surface soil communities found the opposite trend [57]. Nutrient-rich surface communities harbored common soil bacteria found across biomes [62, 63], including a diverse array of trophic levels, such as heterotrophs that degrade plant biomass (e.g., Chthoniobacterales, Desulfuromonadales and Chloroflexi_AD3) [64–66], predatory microbes (e.g., Myxococcales) [59, 62], oligotrophs that can grow in low nutrient conditions (e.g., Ktedonobacterales, Acidobacteriales, Solibacteriales) [25, 62, 63, 65, 67] and lichen symbionts (Chthoniobacterales and Myxococcales) [64], which are common in subarctic surface soils [68]. The higher modularity in the network structure of the surface bacterial communities also indicated more sharing of ecological niches by bacterial taxa in the surface soil [57]. This along with reduced core-dominance (fewer bacterial taxa in high abundance) of these communities, indicate more competition among bacterial taxa, that might have led to the observed reduced diversity in surface communities.

The increased stability of cave environments is reflected in the less variable microbial communities within and between caves. The differentially abundant bacterial taxa in caves tended to be specialists that have also been found in high abundance in other cave ecosystems across geographic regions [26, 69–71]. Knowledge of the ecology of many of these taxa remains limited, but some include chemolithoautotrophs (e.g., Nitrospirales, Nitrosococcales) [72–74] and chemoheterotrophs (e.g., Pirellulales, Blastocatellales) [75–78] that can use inorganic compounds to generate energy. The relatively high abundance of multiple ammonia-oxidising bacterial taxa belonging to the order Nitrosococcales [79] also suggest that these taxa might play key roles through facilitating the nitrogen cycle within these cave ecosystems. The high abundance of microbes associated with nitrogen-based metabolism is a common characteristic of microbiomes adapted to nutrient-poor environments [24, 80]. The presence of slow growing bacterial taxa such as Gemmatimonadales, which specialise on a narrow range of substrates [81], speak to the environmental stability in these caves. The reduced modularity observed in cave bacterial networks, compared to at the surface, along with more interactions between bacterial taxa and the presence of a higher number of abundant bacterial taxa, suggest potential high levels of niche diversification and interspecific dependencies for nutrient cycling among community members. This ecological diversity among bacterial taxa could have also led to the lack of a signal of environmental filtering of cave bacterial communities. Different nutrients can act as selection pressures for different groups of bacterial taxa, implying that there is no single major environmental variable that drives the structure of the whole community, but a combination of multiple drivers (e.g., different nutrients) influencing different components of the community.

### *The paradox of the plankton in bacterial communities of subarctic caves*

Our findings of increased bacterial richness and higher core dominance levels in nutrient-poor cave soils align with the “paradox of the plankton”, originally described by Hutchinson [82]. The paradox describes how limited resources in an area may support an unusually high diversity. Many mechanisms have been proposed to explain the paradox, most of which revolve around aspects of instability [83]. Cave environments, however, offer relatively high levels of stability compared to surface environments in the immediate vicinity. Thus, our results indicate that cave environments with low levels of nutrients require high levels of specialization and co-operation between taxa in order to persevere, and that this in itself leads to higher species richness, which has previously been observed in caves and deep aquifers [84, 85]. Overall, this indicates that the homeostasis of cave bacterial communities depends strongly on environmental stability and hence should be vulnerable to disturbances [57], such as disruptions in natural freeze–thaw cycles at higher latitudes associated with global change. Investigation of bacterial communities solely, however, does not capture the total microbial diversity in these cave ecosystems, as characterization of archaea and fungi are also important to understand cave microbial diversity and ecology [11, 24]. Thus, future studies should include other microbial taxa to thoroughly understand the microbial diversity and nutrient cycling in fragile subarctic cave ecosystems.

## Conclusions

Despite extensive research on surface communities of arctic and subarctic ecosystems, knowledge of biodiversity and ecosystem dynamics in cave habitats in these regions remains sparse. We show, for the first time, that stable subarctic cave environments harbor poor invertebrate faunas, but diverse and complex bacterial microbiomes. This emphasizes that oligotrophic caves with stable environments allow for the development of ecologically diverse bacterial communities with strong interdependencies among them for nutrient cycling. The strong influence of environmental stability on structuring cave bacterial communities indicates increased susceptibility to alterations of natural environmental rhythms (e.g., changes in freeze–thaw cycles) that could distort the stability of these fragile subarctic cave ecosystems. Therefore, conservation and environmental monitoring efforts should include these understudied cave microbial communities. 

## Supplementary Information

**Additional file 1**. This file includes supplementary tables S1, S5, S6, S7, S8, S9, S10, S11, S12, and supplementary figures S1, S2, S3.

**Additional file 2**. ASV table with all the samples (including triplicates) from four cave zones of the six subarctic caves. GenBank accession numbers are given above each column (Table S2).

**Additional file 3.** ASV table with averaged microbiomes for each zone within each cave (averages of triplicates). Only the ASVs with more than 20 sequences are given within the table (Table S3).

**Additional file 4**. Multiple properties of soil samples measured from different cave zones in the six sampled caves (Table S4).

## Data Availability

The data generated and analysed herein are available from the GenBank SRA database (PRJNA669619) and accession number of each sample is given in Additional file 2: Table S2.
