# Supplementary material for: Nutrient-limited subarctic caves harbour more diverse and complex bacterial communities than their surface soil
Source: Environ Microbiome. 2022 Aug 8;17:41. doi: 10.1186/s40793-022-00435-z (PMC9361705; doi:10.1186/s40793-022-00435-z)
Supplement: Supplementary file 1 — Additional file 1. This file includes supplementary tables S1, S5, S6, S7, S8, S9, S10, S11, S12, and supplementary figures S1, S2, S3. [file 40793_2022_435_MOESM1_ESM.docx]

**Supplementary information**

**Nutrient-limited subarctic caves harbour more diverse and complex bacterial communities than their surface soil**

Ana Sofia Reboleira^1,2†*^, Kasun H. Bodawatta^1,2†^, Nynne M.R. Ravn^2^, Stein-Erik Lauritzen^3,4^, Rannveig Øvrevik Skoglund^5^, Michael Poulsen^6^, Anders Michelsen^7^, Knud Andreas Jønsson^2^

^1^ Centre for Ecology, Evolution and Environmental Changes (cE3c), and Departamento de Biologia Animal, Faculdade de Ciências, Universidade de Lisboa, Lisbon, Portugal

^2^ Natural History Museum of Denmark, University of Copenhagen, Universitetsparken 15, 2100 Copenhagen East, Denmark

^3^ Department of Earth Science, University of Bergen, Allegt. 41, 5007 Bergen, Norway

^4^ Centre for Ecological and Evolutionary Synthesis (CEES), Department of Biosciences, University of Oslo, 0316 Oslo, Norway

^5^ Department of Geography, University of Bergen, Fosswinckels gt. 6, 5007 Bergen, Norway

^6^ Section for Ecology and Evolution, Department of Biology, University of Copenhagen, Universitetsparken 15, 2100 Copenhagen East, Denmark

^7^ Section for Terrestrial Ecology, Department of Biology, University of Copenhagen, Universitetsparken 15, 2100 Copenhagen East, Denmark

*Corresponding author: [asreboleira@fc.ul.pt](mailto:asreboleira@fc.ul.pt)

^†^ Equally contributing authors

**Supplementary tables and figures**

**Table S1**. Sampling site variables for the caves around Elgfjellet in Central Norway. Cave entrance orientation (West: W, East: E, South: S) and entrance sizes (large: L, medium: M, small: S) are given within the table.

| Cave | Entrance orientation | Entrance size | Depth (m) | Length (m) | Elevation (m) | Cave Temperature: average during sampling period (ºC) | Latitude | Longitude |
| --- | --- | --- | --- | --- | --- | --- | --- | --- |
| Bjørnetanngrotta | W and vertical | L | 23 | 314 | 622 | 7.7 | 65.37 | 13.16 |
| Spisestuehullet | W and vertical | S | 39 | 295 | 645 | 2.2 | 65.37 | 13.16 |
| Elgfjellhola | E and horizontal | S | 26 | 281 | 594 | 3.1 | 65.37 | 13.16 |
| Pustehola | S | S | 35 | 420 | 600 | 5.9 | 65.37 | 13.17 |
| Brungørrgrotta | S | M | 39 | 352 | 597 | 2.7 | 65.37 | 13.17 |
| Moskusgrotta | E and vertical | M | 25 | 281 | 593 | 2.5 | 65.37 | 13.17 |

**Table S2.** ASV table with all the samples (including triplicates) from four cave zones of the six subarctic caves. GenBank accession numbers are given above each column (Table is in a separate file).

**Table S3.** ASV table with averaged microbiomes for each zone within each cave (averages of triplicates). Only the ASVs with more than 20 sequences are given within the table (Table is in a separate file).

**Table S4.** Multiple properties of soil samples measured from different cave zones in the six sampled caves (Table is in a separate file).

**Table S5.** Results of comparisons of soil properties between different cave zones. Statistical analyses were conducted using one-way ANOVAs when data was homogeneous. If the data was heterogeneous, data were transformed (e.g., log, squire root) to achieve homogeneity. If the achievement of homogeneity was not possible through transformation, we conducted non-parametric Kruskal-Wallis (KW) tests. Significant results are indicated with bold font, while marginally significant results are indicated with an asterisk.

|  | Variance | Data transformation | Test | Test statitisics | p |
| --- | --- | --- | --- | --- | --- |
| Water content | Homogeneous | N/A | ANOVA | F_df_ = 1.564_3,20_ | 0.2291 |
| pH | Heterogeneous | N/A | KW | χ^2^_df_ = 7.478_3_ | 0.0581* |
| **Soil organic matter (SOM)** | **Homogeneous** | **N/A** | **ANOVA** | **F_df_ = 3.972_3,20_** | **0.0226** |
| **%N** | **Homogeneous** | **Squire Root** | **ANOVA** | **F_df_ = 3.461_3,20_** | **0.0358** |
| **%C** | **Homogeneous** | **Log** | **ANOVA** | **F_df_ = 7.320_3,20_** | **0.0017** |
| C:N | Homogeneous | N/A | ANOVA | F_df_ = 0.1021_3,20_ | 0.9580 |
| Inorganic N | Heterogeneous | N/A | KW | χ^2^_df_ = 0.3673_3_ | 0.9469 |
| Dissolved organic nitrogen (DON) | Homogeneous | N/A | ANOVA | F_df_ = 2.606_3,20_ | 0.0801* |
| **Dissolved organic carbon (DOC)** | **Homogeneous** | **Log** | **ANOVA** | **F_df_ = 4.915_3,20_** | **0.0102** |
| PO_4_ | Homogeneous | Log | ANOVA | F_df_ = 2.966_3,20_ | 0.0567* |
| NO_3_ | Homogeneous | N/A | ANOVA | F_df_ = 1.731_3,20_ | 0.1930 |
| NH_4_ | Homogeneous | N/A | ANOVA | F_df_ = 2.051_3,20_ | 0.1391 |
| Conductivity | Homogeneous | N/A | ANOVA | F_df_ = 0.3391_3,20_ | 0.7971 |
| Microbial bound nitrogen (Mic N) | Homogeneous | Log | ANOVA | F_df_ = 0.7711_3,20_ | 0.5240 |
| Microbial bound carbon (Mic C) | Homogeneous | Log | ANOVA | F_df_ = 0.2061_3,20_ | 0.1381 |
| Microbial bound C:N ratio (Mic C:N) | Homogeneous | N/A | ANOVA | F_df_ = 2.189_3,20_ | 0.1210 |
| Microbial bound phosphorus (Mic P) | Heterogeneous | N/A | KW | χ^2^_df_ = 6.423_3_ | 0.0928 |

**Table S6.** Pairwise comparisons of soil properties between different soil zones. Comparisons are given only for overall significant parameters based on Table S5. Significant results are indicated with bold font, while marginally significant results are indicated with an asterisk.

| Soil Property | Post-hoc test | Comparison | p adjusted |
| --- | --- | --- | --- |
| Soil organic matter (SOM) | Tukey HSD | **Surface vs. Twilight** | **0.0393** |
|  |  | **Surface vs. Middle** | **0.0481** |
|  |  | Surface vs. Deep | 0.0605* |
|  |  | Twilight vs. Middle | 0.9966 |
|  |  | Twilight vs. Deep | 0.9966 |
|  |  | Middle vs. Deep | 0.9995 |
|  |  |  |  |
| Nitrogen % | Tukey HSD | Surface vs. Twilight | 0.0567* |
|  |  | Surface vs. Middle | 0.0567* |
|  |  | Surface vs. Deep | 0.1239 |
|  |  | Twilight vs. Middle | 1.000 |
|  |  | Twilight vs. Deep | 0.9769 |
|  |  | Middle vs. Deep | 0.9769 |
|  |  |  |  |
| Carbon % | Tukey HSD | **Surface vs. Twilight** | **0.0033** |
|  |  | **Surface vs. Middle** | **0.0039** |
|  |  | **Surface vs. Deep** | **0.0172** |
|  |  | Twilight vs. Middle | 0.9999 |
|  |  | Twilight vs. Deep | 0.8832 |
|  |  | Middle vs. Deep | 0.9085 |
|  |  |  |  |
| Dissolved organic carbon (DOC) | Tukey HSD | **Surface vs. Twilight** | **0.0185** |
|  |  | **Surface vs. Middle** | **0.0181** |
|  |  | Surface vs. Deep | 0.3807 |
|  |  | Twilight vs. Middle | 0.9999 |
|  |  | Twilight vs. Deep | 0.3847 |
|  |  | Middle vs. Deep | 0.3793 |

**Table S7.** Invertebrate fauna and their abundances in the six studied caves. Juv: juvenile.

| Cave | Elgfjellhola | Moskusgrotta | Bjørnetanngrotta | Pustehola | Spisestuehullet | Brown Stains |
| --- | --- | --- | --- | --- | --- | --- |
| Tardigrada |  |  |  |  |  |  |
| *Hypsibius* cf. *dujardini* (Doyère, 1840) |  |  | 1 |  |  |  |
| Araneae |  |  | 1 juv |  |  |  |
| Linyphiidae |  |  |  |  |  |  |
| Diptera |  |  |  |  |  |  |
| *Trichocera regelationis* (Linnaeus, 1758) | 6 | 23 | 27 | 5 | 7 |  |
| Mycetophilidae |  |  | 1 |  |  |  |
| Coleoptera |  |  |  |  |  |  |
| Staphylinidae |  |  |  |  |  |  |
| *Lesteva pubescens* Mannerheim, 1830 |  |  | 1 |  |  |  |
| *Arpedium quadrum* (Gravenhorst, 1806) |  |  | 17 |  |  |  |

**Table S8.** Dunn’s post-hoc comparisons of observed ASV richness, Chao1 richness estimate and core dominance in different soil communities. Significant differences are indicated with bold font while marginally significant differences are indicated with asterisk.

| Comparison | Observed ASV richness | | Chao 1 richness estimate | | Core dominance | |
| --- | --- | --- | --- | --- | --- | --- |
|  | Test statistics | Adjusted p value | Test statistics | Adjusted p value | Test statistics | Adjusted p value |
| Surface vs. Twilight zone | **-3.328** | **0.0052** | **-3.271** | **0.0031** | **-2.817** | **0.0145** |
| Surface vs. Cave middle | **-2.573** | **0.0303** | **-2.449** | **0.0429** | **-3.225** | **0.0076** |
| Surface vs. Deep cave | **-2.266** | **0.0469** | **-2.245** | **0.0495** | -2.123 | 0.0675* |
| Twilight zone vs. Cave middle | 0.7554 | 0.5399 | 1.021 | 0.3689 | -0.4082 | 0.6831 |
| Twilight zone vs. Deep cave | 1.062 | 0.4326 | 1.225 | 0.3311 | 0.6941 | 0.5852 |
| Cave middle vs. Deep cave | 0.3063 | 0.7594 | 0.2041 | 0.8383 | 1.102 | 0.4055 |

**Table S9.** Results of linear regressions investigating the association between different soil properties and ASV diversity in soil samples. Significant associations are indicated with bold font, while marginally significant associations are indicated with an asterisk. In SOM we removed one outlier from the surface communities.

| Soil parameter | F_df_ | Adjusted R^2^ | p |
| --- | --- | --- | --- |
| **pH** | **18.96_1, 22_** | **0.4384** | **0.0003** |
| Soil water content | 3.824_1,22_ | 0.1094 | 0.0633* |
| **Soil organic matter (SOM)** | **7.651_1,21_** | **0.2321** | **0.0116** |
| Nitrogen % | 2.525_1,22_ | 0.0622 | 0.1263 |
| Carbon % | 2.321_1,22_ | 0.0543 | 0.1419 |
| C:N ratio | 0.0939_1,22_ | -0.0411 | 0.7621 |
| Dissolved organic nitrogen (DON) | 2.542_1,22_ | 0.0628 | 0.1252 |
| Dissolved organic carbon (DOC) | 3.255_1,22_ | 0.0893 | 0.0849* |
| **PO**$\begin{matrix} \mathbf{-3} \\ \mathbf{4} \end{matrix}$ **(Phosphate)** | **4.827**_1,22_ | **0.1427** | **0.0388** |
| NO$\begin{matrix} - \\ 3 \end{matrix}$(Nitrate) | 0.0342_1,22_ | -0.0438 | 0.8549 |
| NH$\begin{matrix} + \\ 4 \end{matrix}$ (Ammonium) | 2.858_1,22_ | 0.0748 | 0.1051 |
| Inorganic nitrogen | 1.001_1,22_ | 0.0002 | 0.3281 |
| **Microbial bound carbon (MicC)** | **4.366_1,22_** | **0.1277** | **0.0484** |
| Microbial bound nitrogen (MicN) | 1.547_1,22_ | 0.0232 | 0.2267 |
| **Microbial bound C:N ratio (Mic C:N)** | **9.796_1,22_** | **0.2766** | **0.0049** |

**Table S10.** Pairwise comparisons (pair wise adonis 10,000 permutations) of microbiome community levels differences (based on Bray-Curtis distances) between different zones.

| Comparison | F | R^2^ | Adjusted p value |
| --- | --- | --- | --- |
| Surface vs. Twilight | 3.784 | 0.2745 | 0.0162 |
| Surface vs. Middle | 3.832 | 0.2771 | 0.0084 |
| Surface vs. Deep | 3.629 | 0.2663 | 0.01439 |

**Table S11**. Results of the envfit analysis indicating soil properties that are significantly associated with bacterial community composition differences in surface and cave zones. Significant associations are indicated with bold font, while marginally significant associations are indicated with an asterisk.

| Soil property | NMDS1 | NMDS2 | R^2^ | p value |
| --- | --- | --- | --- | --- |
| **pH** | **-0.9217** | **-0.3632** | **0.7555** | **0.0011** |
| **Soil water content** | **-0.1998** | **0.9798** | **0.3325** | **0.0301** |
| Soil organic matter (SOM) | 0.5722 | -0.8201 | 0.3016 | 0.0631* |
| **Nitrogen %** | **0.4603** | **-0.8878** | **0.3717** | **0.0301** |
| **Carbon %** | **0.4103** | **-0.9119** | **0.3711** | **0.0250** |
| Dissolved organic nitrogen (DON) | 0.2601 | -0.9656 | 0.2596 | 0.0932 |
| **Dissolved organic carbon (DOC)** | **0.2434** | **-0.9699** | **0.3170** | **0.0431** |
| **PO**$\begin{matrix} \mathbf{-3} \\ \mathbf{4} \end{matrix}$ **(Phosphate)** | **0.1565** | **-0.9877** | **0.3652** | **0.0212** |
| NO$\begin{matrix} - \\ 3 \end{matrix}$(Nitrate) | 0.2275 | -0.9738 | 0.1925 | 0.1611 |
| NH$\begin{matrix} + \\ 4 \end{matrix}$ (Ammonium) | -0.0003 | -1.000 | 0.2174 | 0.1233 |
| Inorganic nitrogen | 0.1013 | -0.9945 | 0.2318 | 0.1161 |
| **Microbial bound carbon (MicC)** | **0.3891** | **-0.9212** | **0.4634** | **0.0080** |
| Microbial bound nitrogen (MicN) | 0.2773 | -0.9608 | 0.3059 | 0.0611* |
| **Microbial bound C:N ratio (Mic C:N)** | 0.9412 | 0.3379 | 0.1876 | 0.1610 |

**Table S12**. Overall relative abundance (in % ± SD) of Major bacterial phyla in different zones of caves.

| Phyla | Surface | Twilight | Middle | Deep |
| --- | --- | --- | --- | --- |
| Proteobacteria | 28.8% ± 8.8% | 33.3% ± 5.4% | 33.8% ± 5.0% | 35.6% ± 9.8% |
| Acidobacteria | 20.4% ± 3.0% | 16.6% ± 2.4% | 17.7% ± 4.7% | 16.5% ± 7.8% |
| Actinobacteria | 10.4% ± 6.8% | 11.4% ± 2.1% | 10.0% ± 1.6% | 8.2% ± 2.6 |
| Chloroflexi | 10.3% ± 5.6% | 7.8% ± 1.9% | 6.4% ± 2.2% | 7.1% ± 2.3% |
| Planctomycetes | 4.4% ± 1.5% | 7.1% ± 1.0% | 8.5% ± 2.2% | 8.0% ± 2.0% |
| Bacteroidetes | 3.4% ± 1.3% | 6.0% ± 0.9% | 5.4% ± 1.3% | 5.4% ± 1.5% |
| Verrucomicrobia | 11.5% ± 7.0% | 2.9% ± 1.2% | 3.5% ± 2.1% | 2.8% ± 1.1% |
| Gemmatimonadetes | 2.7% ± 2.2% | 4.0% ± 1.1% | 3.8% ± 2.9% | 4.1% ± 1.5% |
| Rokubacteria | 3.4% ± 1.0% | 1.7% ± 1.6% | 2.4% ± 1.3% | 2.1% ± 1.6% |
| Patescibacteria | 0.8% ± 0.4% | 2.6% ± 2.1% | 1.5% ± 0.6% | 1.7% ± 1.3% |

**Supplementary figures**

**Fig. S1.** NMDS plot showing the bacterial community similarities (Bray-Curtis distances) among triplicate samples from different sampling localities in caves (stress = 0.0907). Colours represent the different caves, while symbols represent the sampling zones.

Fig. S2. Distribution of the number of interactions (degree) that ASVs have in each network. Stability threshold and the sparsity of each network is given within appropriate graph.

Fig S3. Box plots demonstrating the Nearest Taxon Index (NTI – average phylogenetic distance between two taxa within a community) and the βNTI (average nearest phylogenetic distances of taxa between communities) of bacterial communities in four sampling zones. Lower case letters on top of box plots indicate the outcome of the Dunn’s post-hoc tests.
